# Efficacy of AAV9-mediated *SGPL1* gene transfer in a mouse model of S1P lyase insufficiency syndrome

**DOI:** 10.1172/jci.insight.145936

**Published:** 2021-04-22

**Authors:** Piming Zhao, Gizachew B. Tassew, Joanna Y. Lee, Babak Oskouian, Denise P. Muñoz, Jeffrey B. Hodgin, Gordon L. Watson, Felicia Tang, Jen-Yeu Wang, Jinghui Luo, Yingbao Yang, Sarah King, Ronald M. Krauss, Nancy Keller, Julie D. Saba

**Affiliations:** 1 Department of Pediatrics, UCSF, San Francisco, California, USA.; 2 Cure Genetics, Suzhou, China.; 3 Department of Pathology, University of Michigan, Ann Arbor, Michigan, USA.

**Keywords:** Metabolism, Therapeutics, Gene therapy, Genetic diseases

## Abstract

Sphingosine-1-phosphate lyase insufficiency syndrome (SPLIS) is a rare metabolic disorder caused by inactivating mutations in sphingosine-1-phosphate lyase 1 (*SGPL1*), which is required for the final step of sphingolipid metabolism. SPLIS features include steroid-resistant nephrotic syndrome and impairment of neurological, endocrine, and hematopoietic systems. Many affected individuals die within the first 2 years. No targeted therapy for SPLIS is available. We hypothesized that *SGPL1* gene replacement would address the root cause of SPLIS, thereby serving as a universal treatment for the condition. As proof of concept, we evaluated the efficacy of adeno-associated virus 9–mediated transfer of human *SGPL1* (AAV-SPL) given to newborn *Sgpl1*-KO mice that model SPLIS and die in the first weeks of life. Treatment dramatically prolonged survival and prevented nephrosis, neurodevelopmental delay, anemia, and hypercholesterolemia. STAT3 pathway activation and elevated proinflammatory and profibrogenic cytokines observed in KO kidneys were attenuated by treatment. Plasma and tissue sphingolipids were reduced in treated compared with untreated KO pups. *SGPL1* expression and activity were measurable for at least 40 weeks. In summary, early AAV-SPL treatment prevents nephrosis, lipidosis, and neurological impairment in a mouse model of SPLIS. Our results suggest that *SGPL1* gene replacement holds promise as a durable and universal targeted treatment for SPLIS.

## Graphical abstract

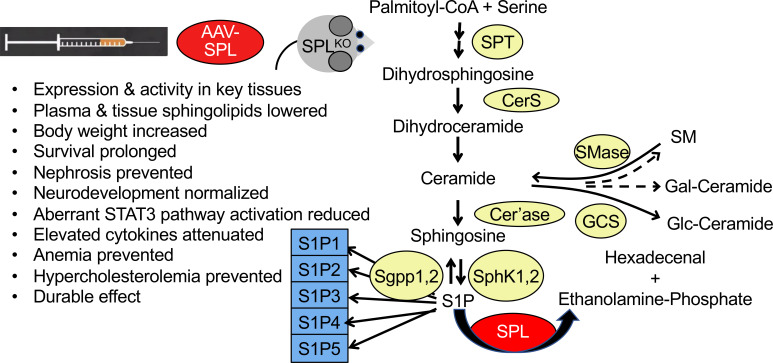

## Introduction

Biallelic loss-of-function mutations in sphingosine-1-phosphate lyase 1 (*SGPL1*) result in sphingosine-1-phosphate lyase insufficiency syndrome (SPLIS), a rare metabolic disorder associated with nonlysosomal sphingolipid storage (1, 2). The syndrome was first described in 2017 (3, 4). About 50 cases have been reported to date (5–10). Most affected individuals exhibit steroid-resistant nephrotic syndrome (SRNS) progressing rapidly to end-stage renal disease. Nephrosis is most often associated with focal segmental glomerulosclerosis pathology, including the aggressive collapsing variant. Primary adrenal insufficiency is the second most common feature. Defects of the central and peripheral nervous systems, which may include developmental delay or regression accompanied by characteristic findings on magnetic resonance imaging, occur with other disease features or independently in about half of cases (1, 11). T cell lymphopenia seems to be a universal feature, although some level of T cell function usually persists. A wide range of severity has been observed, with some affected individuals dying in utero, and others in infancy, while still others have presented later in the first decade of life and are living into adulthood with supportive care. No specific therapies for the treatment of SPLIS have been established.

*SGPL1* encodes sphingosine phosphate lyase (SPL), the enzyme responsible for the final step of sphingolipid metabolism (12). SPL catalyzes the irreversible degradation of phosphorylated sphingoid bases, generating 2 products: a long chain aldehyde and ethanolamine phosphate. The bioactive sphingolipid sphingosine-1-phosphate (S1P) is the major SPL substrate. S1P is the ligand for G protein–coupled S1P receptors (S1PRs) involved in the control of actin cytoskeletal organization, cell migration, and cell survival (13). S1P signaling regulates lymphocyte trafficking, angiogenesis, inflammation, and other physiological processes (14). SPL inactivation in vivo causes profound tissue S1P elevation and accumulation of upstream sphingolipid intermediates, such as ceramide and sphingosine, which have cytotoxic properties (15). SPL inactivation disrupts S1P chemotactic gradients needed for lymphocyte egress from lymphoid tissues, which explains why individuals with SPLIS are lymphopenic (16, 17). The mechanisms responsible for the pathological impact of SPL inactivation on organ functions could be due to aberrant S1P signaling, intracellular effects of S1P, accumulation of cytotoxic sphingolipids, deficiency of SPL products, broader disruption of lipid homeostasis, or any combination of these.

SPL localizes to the outer membrane of the endoplasmic reticulum (18). In contrast to conventional sphingolipidoses — which are usually lysosomal lipid storage conditions — SPLIS shows no evidence of lysosomal lipid storage. The first SPLIS cases were identified by next-generation sequencing (NGS). Individuals with SPLIS have since been identified by NGS or disease-focused diagnostic genetic panels. Pathogenic variants including missense, nonsense, and splice site mutations affecting 14 of the 15 *SGPL1* exons have been reported (19).

We hypothesized that virus-mediated gene replacement may afford a practical strategy to treat and potentially cure SPLIS by specifically addressing its root cause. Adeno-associated virus (AAV) is a small, single-stranded DNA virus belonging to the parvovirus family (20). AAV vectors are nonintegrating, form episomal concatemers, have relatively low immunogenicity, and can persist in postmitotic tissues for years (21, 22). Twelve serotypes are recognized based on neutralizing antibodies against capsid proteins that determine tissue tropism (22). Safety of AAV-based gene therapy has been established in more than 170 clinical trials, with proven efficacy for several diseases. In 2019, the FDA approved Zolgensma, the first systemic, AAV-mediated gene therapy for spinal muscular atrophy (23).

A constitutive *Sgpl1*-KO mouse generated by gene trapping represents the most well-established murine model of SPLIS (24). *Sgpl1*-KO pups are born at 25% frequency from heterozygous mating pairs. *Sgpl1*-KO pups lack SPL expression and activity and accumulate sphingolipids (15). They exhibit runting at about a week of life accompanied by anemia and lymphopenia and die at the time of weaning (around 3 weeks of age). They develop nephrosis associated with hypoalbuminemia and elevated urine albumin/creatinine ratio (ACR) (25). Histological examination of kidney sections revealed enlarged glomeruli and ultrastructural evidence of podocyte foot process effacement (3, 25). Subtle abnormalities of adrenal gland histology have been observed (4). Overt neurological defects have not been reported in *Sgpl1*-KO mice; however, brain-specific *Sgpl1*-KO mice that survive to adulthood exhibit behavioral and motor functional deficits (26). Thus, *Sgpl1*-KO mice recapitulate many biochemical and phenotypic features of SPLIS. Other features of the KO include high liver and plasma cholesterol, glycerophospholipids, and Th-17 pathway activation (15, 27). Heterozygous *Sgpl1*-null mice are indistinguishable from WT in growth, survival, and sphingolipid levels. Further, transgenic mice with 10%–20% of WT SPL levels are lymphopenic but otherwise healthy, live normal life spans, and do not exhibit the severe phenotypes found in *Sgpl1*-KO mice (28). Thus, a modest level of SPL expression may prevent most SPLIS-related pathologies.

In this study, we used AAV9-mediated gene delivery to transfer the WT human *SGPL1* cDNA to the tissues of *Sgpl1*-KO mice within the first days of life (DOL). Our baseline studies reveal for the first time to our knowledge that *Sgpl1*-KO mice exhibit delayed acquisition of neurodevelopmental milestones and are deficient in corticosterone, the main glucocorticoid found in mice. We demonstrate that *SGPL1* gene replacement prevents nephrosis, developmental delay, and lipidosis in *Sgpl1*-KO mice and dramatically prolongs their survival. Further, we observed elevated proinflammatory and profibrogenic cytokines and activation of the STAT3 signaling pathway in untreated KO kidneys, all of which were attenuated by treatment. Our cumulative results provide proof of concept for AAV-mediated *SGPL1* gene transfer as a potentially curative therapy for SPLIS and provide insight into the possible pathomechanism of SPLIS nephrosis.

## Results

### AAV-SPL restores SPL expression and activity in SPLIS patient–derived fibroblasts.

**Figure 1:**
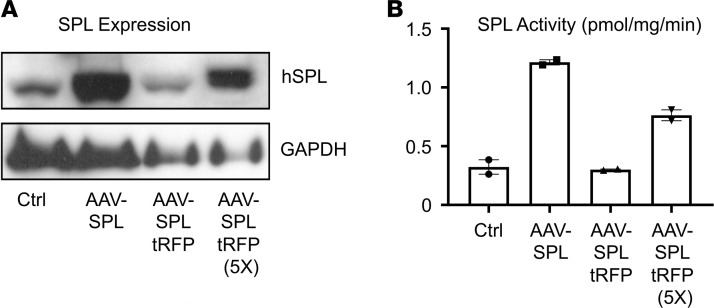
AAV-SPL expression and activity in vitro. The human *SPL* (hSPL) cDNA and the hSPL-tRFP cDNA were cloned into pAAV-MCS, packaged in AAV8, and used to transduce SPLIS skin fibroblasts. (**A**) Immunoblot of hSPL in whole cell extracts of SPLIS fibroblasts treated with vehicle (Ctrl), AAV-SPL, an equal volume of AAV-SPLtRFP, or a 5-fold higher volume of AAV-SPL-tRFP. (**B**) SPL activity in extracts corresponding to the samples described in **A**.

Human WT *SGPL1* cDNA or a self-cleaving bicistronic system for coexpressing red fluorescent protein (RFP) and SPL were cloned in an AAV2 vector under control of the CMV promoter. The resulting constructs, AAV-SPL and AAV-SPL-tRFP, were amplified, packaged in AAV8 capsid, and used to transduce SPLIS patient–derived fibroblasts, which exhibit low SPL expression and activity (10). Transduction of fibroblasts with AAV-SPL increased SPL expression (Figure 1A) and activity (Figure 1B). Fibroblasts transduced with AAV-SPL-tRFP resulted in lower expression and activity. Based on these results, AAV-SPL was chosen for validation in vivo. A construct expressing an SPL that harbors a missense mutation at lysine 353 (AAV-SPL^K353L^) — which is the site for pyridoxal 5′-phosphate cofactor binding and was shown to completely eliminate enzyme function — was generated to serve as a biochemical control. AAV9 was selected for in vivo studies based on its broad tropism, including brain, adrenal gland, and kidney — all of which are involved in SPLIS — as well as liver, a major site of metabolism of blood sphingolipids.

### Treatment of Sgpl1-KO mice with AAV-SPL dramatically prolongs survival.

**Figure 2:**
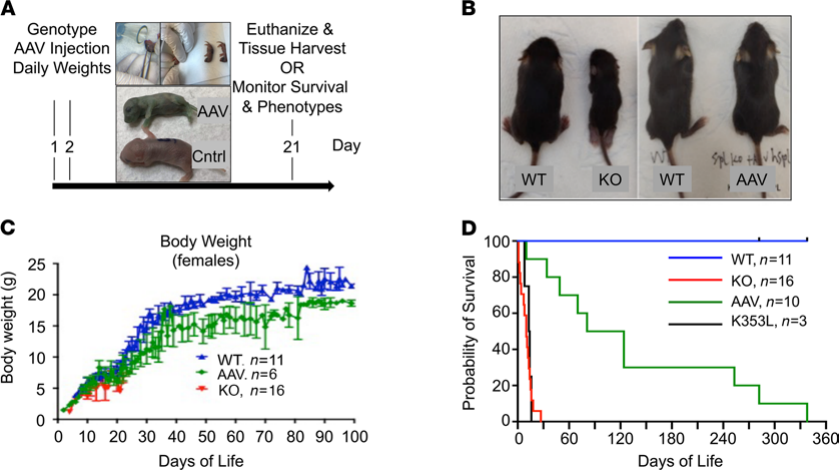
Treatment of newborn *Sgpl1*-KO mice with AAV-SPL prolongs survival. (**A**) Treatment schedule, with upper images showing injection site and lower image showing green color of properly injected KO pup (AAV) next to untreated WT pup (Cntrl). (**B**) Images showing size discrepancy between WT and untreated *Sgpl1*-KO littermates at 17 DOL versus similarity in size of WT and AAV-SPL–treated *Sgpl1*-KO (AAV) littermates at 22 DOL. (**C**) Weight gain of female WT (blue), untreated *Sgpl1*-KO (red), and AAV-SPL–treated KO (green) mice. (**D**) Kaplan-Meier survival curve for WT (blue), untreated *Sgpl1*-KO (red), AAV-SPL–treated KO (green), and AAV-SPL^K353L^–treated KO (black) mice. Log-rank test with Bonferroni’s correction: for WT vs. all other groups, *P* < 0.0003; AAV vs. KO, *P* < 0.0003; KO vs. K353L, no significant difference (NSD).

To test the impact of *SGPL1* gene replacement on *Sgpl1*-KO mouse survival, AAV-SPL was delivered to 10 newborn *Sgpl1*-KO pups using the strategy shown in Figure 2A. Litters produced from heterozygous matings were genotyped on DOL 1 by toe biopsy. *Sgpl1*-KO pups were treated at 1–2 DOL with a single dose of approximately 7 × 10^11^ vector genomes (vg) by intravenous (i.v.) injection. An additional 3 KO pups were injected with the same dose of AAV-SPL^K353L^. Pups were monitored for weight gain and euthanized when the humane endpoint was reached. As shown in Figure 2B, AAV-SPL–treated *Sgpl1*-KO mice were appreciably larger than untreated *Sgpl1*-KO mice. *Sgpl1*-KO mice treated with AAV-SPL gained weight steadily, as shown in Figure 2C. Mean survival increased from 11 ± 7 days in untreated KO controls to 137 ± 114 days in treated mice (*n* = 16 untreated, *n* = 10 treated; *P* < 0.0002), with some treated mice living for 8–11.5 months (Figure 2D). In contrast, mice treated with catalytically inactive AAV-SPL^K353L^ showed a mean survival of 12.8 ± 6.2 days (Figure 2D). Based on these findings, we concluded that introduction of a functional hSPL into *Sgpl1*-KO pups significantly prolongs their survival.

### AAV-SPL treatment of Sgpl1-KO mice prevents SPLIS-associated nephrosis.

**Figure 3:**
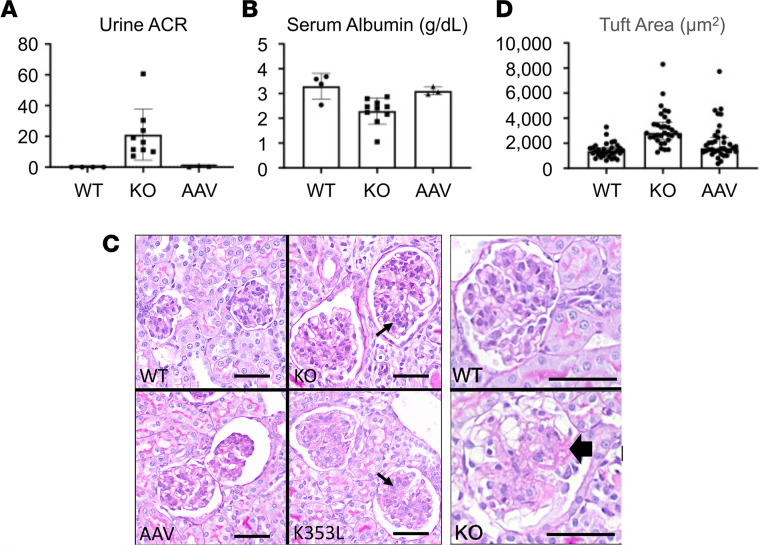
AAV-SPL treatment prevents development of SPLIS nephrosis. (**A**) Urine ACR in WT (*n* = 4), KO (*n* = 9), and AAV-SPL–treated KO (AAV, *n* = 3) mice. (**B**) Serum albumin in WT (*n* = 4), KO (*n* = 10), and AAV-SPL–treated KO (*n* = 3) mice. For **A** and **B**, unpaired *t* test was performed with Welch’s correction when appropriate and Bonferroni’s corrections for comparisons of experimental groups with WT. For **A**, *P* = 0.01 for KO vs. WT. For **B**, *P* = 0.015. For **A** and **B**, there was NSD between AAV vs. WT. (**C**) Quadrants show kidney sections stained with periodic acid–Schiff from WT, KO, AAV-SPL–treated KO (AAV), and AAV-SPL^K353L^–treated KO (K353L) mice. KO and K353L kidney sections show enlarged glomeruli with mesangial expansion, not seen in sections of WT and AAV mice. Small arrows indicate mesangial expansion by cells and matrix. White areas surrounding glomeruli are fixation artifact. Scale bar: 50 μm. Image to right shows sclerosis in KO glomerulus (black arrowhead), with a WT glomerulus shown above it for contrast. (**D**) Glomerular tuft area, which includes the capillaries, mesangium, and podocytes and not Bowman’s space or capsule. Mann-Whitney *U* test was performed with Bonferroni’s correction. For KO vs. WT, *P* < 0.0002. There was NSD between AAV vs. WT.

Renal involvement is present in 80% of SPLIS cases and is a major cause of morbidity, hospitalizations, surgical intervention (renal transplantation), and death. Similarly, *Sgpl1*-KO mice develop nephrosis with high urine albumin/creatinine ratio (ACR) and low serum albumin levels, accompanied by pathological changes consistent with podocyte and glomerular injury prior to their demise at the time of weaning (at 21 DOL). The first few *Sgpl1*-KO mice treated in our series lived for 35–70 days and exhibited higher serum albumin levels than untreated *Sgpl1*-KO mice (data not shown). This indicated that AAV-SPL treatment may have delayed the progression of nephrosis but did not completely prevent it. In contrast, once we optimized our virus delivery, we could routinely prolong survival of treated mice for 8–11.5 months. These mice showed normal ACR (Figure 3A) and serum albumin levels (Figure 3B) up to the time of their deaths. Periodic acid–Schiff–stained sections of kidney cortices from untreated *Sgpl1*-KO mice showed enlarged glomeruli with a wide size distribution and mesangial expansion compared with WT glomeruli (Figure 3C). These features were absent in the kidney cortices of *Sgpl1*-KO mice treated with AAV-SPL but were present in those of *Sgpl1*-KO mice treated with catalytically inactive AAV-SPL^K353L^ (Figure 3C). Average glomerular tuft size is shown quantitatively in Figure 3D. Some glomeruli of untreated *Sgpl1*-KO mice and *Sgpl1*-KO mice treated with AAV-SPL^K353L^ exhibited sclerosis, an example of which is shown in Figure 3C. No glomeruli from WT and AAV-SPL–treated *Sgpl1*-KO mice exhibited focal sclerosis.

### SPLIS kidneys show Stat3 activation and cytokine upregulation.

**Figure 4:**
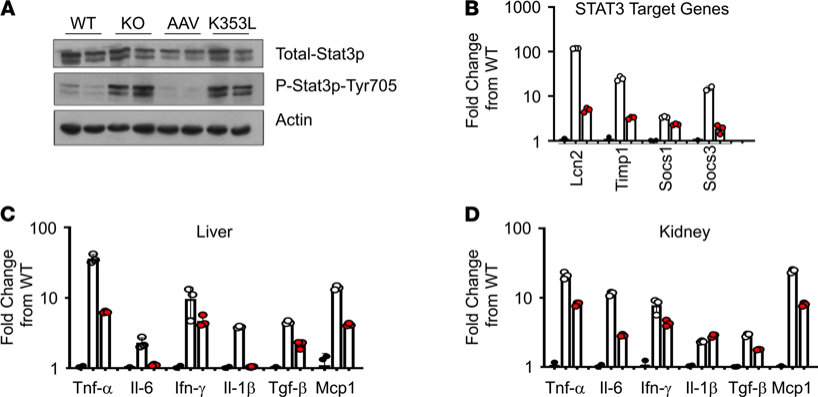
Stat3 activation and cytokine upregulation in SPLIS kidneys. (**A**) Immunoblot showing total and tyrosine 705–phosphorylated (activated) Stat3 in kidneys of WT, KO, AAV-SPL–treated KO (AAV), and AAV-SPL^K353L^–treated KO (K353L) mice; *n* = 2/group. GAPDH is a loading control. (**B**) Relative expression of Stat3 target genes *Lcn2*, *Timp1*, and *Socs1* and *Socs3* in WT (black circles), KO (white circles), and AAV kidney (red circles), shown as fold change from WT. For KO vs. AAV, *P* = 0.003 for all genes except *Socs3*. (**C**) Liver and (**D**) kidney cytokines of WT, KO, and AAV mice, shown as log fold change from WT, with same key as in **B**. For **B**–**D**, unpaired *t* test with Bonferroni’s correction (and Welch’s correction where appropriate) was applied. For liver cytokines, AAV vs. KO: *P* = 0.0097 for *Tnf-α*; *P* = 0.0363 for *Il-6*; NSD for *Ifn-γ*; *P* = 0.0004 for *Il-1β*; *P* = 0.0003 for *Tgf-β*; *P* < 0.0001 for *Mcp1*. For kidney cytokines, AAV vs. KO: *P* = 0.0009 for *Tnf-α*; *P* = 0.0023 for *Il-6*; *P* = 0.036 for *Ifn-γ*; *P* = 0.0046 for *Il-1β*; *P* = 0.005 for *Tgf-β*; *P* < 0.0001 for *Mcp1*.

STAT3 is a transcription factor that contributes to carcinogenesis and the regulation of inflammation, autophagy, cellular metabolism, and mitochondrial function (29). It can be activated by various cytokines through binding and activation of gp130, the IL-6 receptor. Glomerular injury and S1P signaling have been associated with activation of STAT3 in mice and humans (30–34). We compared the phosphorylated (active) and total mouse Stat3 protein levels in *Sgpl1* WT and KO kidney homogenates by immunoblotting. As shown in Figure 4A, *Sgpl1*-KO kidneys exhibited activation of Stat3 in comparison with WT controls. Stat3 activation was muted in AAV-SPL–treated KO kidneys, whereas kidneys of KO mice treated with AAV-SPL^K353L^ were not protected from Stat3 activation. Expression of Stat3 target genes suppressor of cytokine signaling 1 and 3 (*Socs1*, *Socs3*) and 2 others implicated in various forms of kidney injury, namely lipocalin 2 (*Lcn2*) and tissue inhibitor of metalloprotease 1 (*Timp1*), were measured by quantitative real-time PCR (RT-PCR) in kidney tissues from each group. Stat3 target genes were upregulated in untreated *Sgpl1*-KO kidney tissue compared with WT, while kidneys of AAV-SPL–treated KO mice showed lower Stat3 target gene elevation compared with untreated *Sgpl1*-KO mice (Figure 4B). Proinflammatory cytokine elevation in the liver tissues of *Sgpl1*-KO mice was previously reported (27). We confirmed that the proinflammatory cytokine and STAT3 pathway activator *Il-6* was upregulated in *Sgpl1*-KO liver tissues (Figure 4C). Additional proinflammatory cytokines, including *Tnf-α*, *Ifn-γ*, *Il-1β*, and monocyte chemoattractant protein 1 (*Mcp1*), were elevated in *Sgpl1*-KO liver compared with WT liver (Figure 4C). The cytokine *Tgf-β* is a well-characterized inducer of fibrosis. *Tgf-β* levels were also elevated in *Sgpl1*-KO liver compared with WT liver (Figure 4C). Importantly, each of these cytokines was also elevated in the kidneys of the *Sgpl1*-KO mice compared with WT (Figure 4D). Further, AAV-SPL treatment diminished cytokine levels in *Sgpl1*-KO kidneys (Figure 4D). These findings demonstrate that AAV-SPL treatment prevents the development of nephrosis, Stat3 activation, and proinflammatory and fibrogenic cytokine upregulation in various tissues of *Sgpl1*-KO mice.

### AAV-SPL treatment prevents neurodevelopmental delay in preweaned Sgpl1-KO mice.

**Figure 5:**
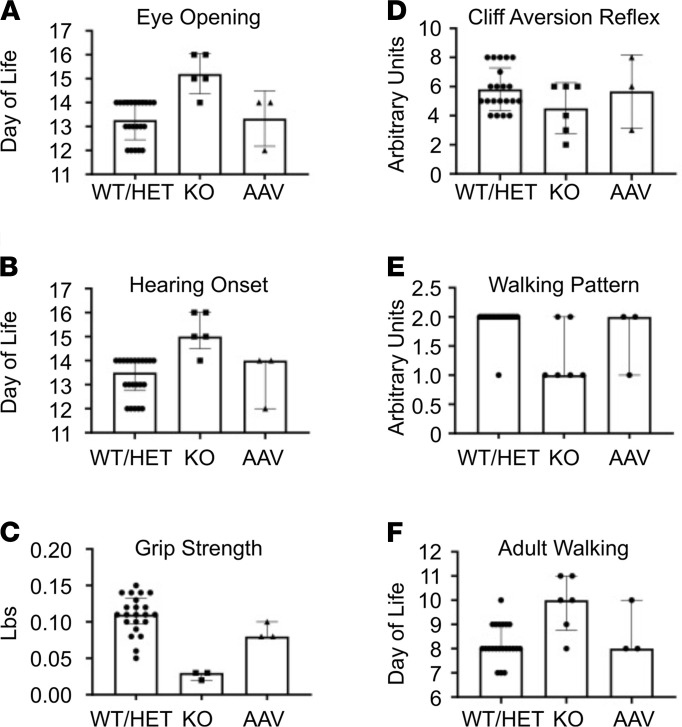
*Sgpl1-*KO mice exhibit developmental delay, which is prevented by AAV-SPL. Six neurodevelopmental milestones were scored in WT (*n* = 8), heterozygous (HET, *n* = 14), KO (*n* = 6), and AAV-SPL–treated KO (AAV, *n* = 3) pups. For **A** and **D**, unpaired *t* test with Bonferroni’s correction was applied. For **B**, **C**, and **F**, Mann-Whitney *U* with Bonferroni’s correction was applied. For KO vs. WT comparisons: (**A**, *P* < 0.0002); (**B**, *P* = 0.001); (**C**, *P* = 0.0008); (**D**, NSD; **E**, *P* = 0.0068); (**F**, *P* = 0.0056). There was NSD between AAV and WT/HET or WT and HET for **A**–**F**.

In nearly half of SPLIS cases, neurological manifestations are present, including sensorineural hearing loss, cranial nerve defects, a Charcot-Marie-Tooth–type peripheral neuropathy, seizures, ataxia, and developmental delay or regression. Brain-specific conditional *Sgpl1*-KO mice live normal life spans but exhibit motor and behavioral abnormalities as adults (26). However, characterization of the neurodevelopmental function of global *Sgpl1*-KO pups has not been reported to our knowledge. A battery of tests designed to measure the achievement of basic neurodevelopmental milestones in preweaned mice was used to compare AAV-SPL–treated and untreated *Sgpl1*-KO pups and WT littermates (Figure 5, A–F). *Sgpl1*-KO pups showed significant delays in the achievement of eye opening, hearing onset, and adult pattern walking. In addition, grip strength was weaker in *Sgpl1*-KO compared with WT pups, indicating a motor function deficit. Cliff aversion reflex scores were lower in KO pups compared with WT littermates, but the difference was not statistically significant. Other tests, including paw grasping reflex, righting reflex, negative geotaxis, and inverted clinging obstacle tests, were either highly variable from day to day or did not reveal defects in the *Sgpl1*-KO pups (data not shown). In contrast, the neurodevelopmental scores in AAV-SPL–treated KO pups were not significantly different from WT (Figure 5, A–F). We infer from these results that neurological impairment is manifest in *Sgpl1*-KO mice as early as the first weeks of life and that immediate treatment after birth with AAV-SPL prevents neurodevelopmental delay.

### Sgpl1-KO mice exhibit glucocorticoid deficiency unresponsive to AAV-SPL.

**Figure 6:**
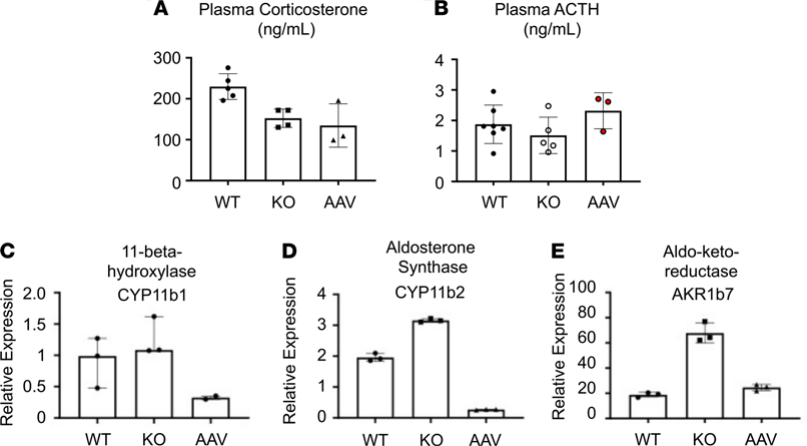
*Sgpl1-*KO mice exhibit glucocorticoid deficiency. (**A**) Corticosterone was measured in plasma of WT (*n* = 5), KO (*n* = 4), and AAV-SPL–treated KO (AAV, *n* = 3) mice. (**B**) ACTH levels were measured in the plasma of WT (*n* = 8), KO (*n* = 5), and AAV-SPL–treated KO (AAV, *n* = 3) mice. There are NSDs among the groups. (**C**–**E**) Expression levels of *Cyp11b1*, *Cyp11b2*, and *Akr1b7* were measured in adrenal gland tissues of WT, KO, and AAV mice (*n* = 3/group except for AAV in **C**, where *n* = 2). For **A**, **C**, and **D**, unpaired *t* test with Bonferroni’s correction was performed (with Welch’s correction in **C**). For **B**, Mann-Whitney *U* was performed. In **A**, KO vs. WT, *P* = 0.009; AAV vs. WT, *P* = 0.035. In **B**, KO vs. WT, NSD; insufficient data for AAV vs. WT. In **C**, KO vs. WT, *P* = 0.0016; AAV vs. WT, *P* = 0.0032. In **D**, KO vs. WT, *P* = 0.001; AAV vs. WT, NSD.

Approximately 70% of patients with SPLIS exhibit glucocorticoid hormone deficiency. However, blood steroid hormone measurements in *Sgpl1*-KO mice have not been hitherto reported to our knowledge. Plasma levels of corticosterone, the main glucocorticoid hormone in mice, were measured in treated and untreated *Sgpl1*-KO mice and WT littermate controls. Samples were collected at the corticosterone peak of the circadian cycle. As shown in Figure 6A, *Sgpl1*-KO mice exhibited significantly lower corticosterone levels than WT levels regardless of AAV-SPL treatment. Thus, *Sgpl1*-KO mice exhibit adrenal insufficiency, which appears refractory to treatment. To determine whether low corticosterone levels in *Sgpl1*-KO mice resulted from disruption of the hypothalamic-pituitary-adrenal axis, we measured plasma levels of the pituitary hormone adrenocorticotropic hormone (ACTH). As shown in Figure 6B, there was a trend toward lower ACTH levels in the KO compared with WT groups, with a restoration of ACTH levels in the AAV-SPL–treated KO group. However, the differences between groups were not significant. To gain additional insight into the impact of AAV-SPL on adrenal gland function, we measured the gene expression of *Cyp11b1*, *Cyp11b2*, and *Akr1b7* — which respectively encode the 3 adrenal cortical enzymes 11-β-hydroxylase, aldosterone synthase, and aldo-keto-reductase — in the adrenal tissues of treated and untreated *Sgpl1*-KO and WT littermate mice (Figure 6, C–E). *Cyp11b1* and *Cyp11b2* are involved in adrenal hormone synthesis (35), whereas *Akr1b7* is involved in lipid peroxide detoxification (36). Interestingly, all 3 genes were upregulated in untreated *Sgpl1*-KO adrenal glands, whereas levels were either suppressed (*Cyp11b1*, *Cyp11b2*) or normalized compared with WT mice in the adrenal glands of AAV-SPL–treated KO mice. We infer from these findings that *Sgpl1*-KO mice exhibit adrenal insufficiency of unclear origin with reactive upregulation of cortical gene expression. *SGPL1* gene therapy reduces adrenal cortical gene expression to WT levels or below but does not restore normal corticosterone levels under the conditions tested.

### AAV-SPL treatment corrects anemia and hypercholesterolemia in Sgpl1-KO mice.

**Figure 7:**
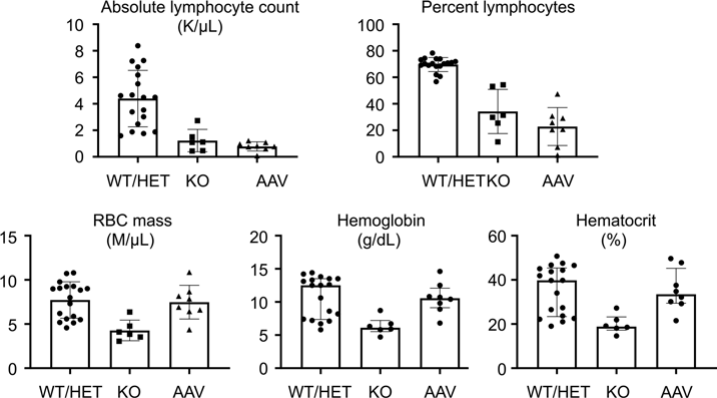
Hematological parameters of treated and untreated *Sgpl1*-KO mice. (**A**) Blood parameters including absolute lymphocyte count (K/μL), percentage lymphocytes (% lymph), RBC mass (M/μL), hemoglobin (g/dL), and hematocrit (%) were measured in WT/HET (*n* = 18), KO (*n* = 6), and AAV-SPL–treated KO (AAV) mice (*n* = 8). All mice were euthanized at 28 DOL. All parameters were evaluated using unpaired 2-tailed *t* test with Bonferroni’s correction. Welch’s correction was applied when appropriate. For absolute lymphocyte count, WT/HET vs. KO, *P* < 0.004; AAV vs. WT/HET, *P* < 0.0002. For percentage lymphocytes, WT/HET vs. KO, *P* = 0.006; AAV vs. WT/HET, *P* <0.0002. For RBC mass, WT/HET vs. KO, *P* = 0.0018. For hemoglobin, WT/HET vs. KO, *P* = 0.0048. For hematocrit, WT/HET vs. KO, *P* = 0.0026. For RBC mass, hemoglobin and hematocrit, AAV vs. WT/HET, there was NSD.

Peripheral blood counts in *Sgpl1*-KO mice revealed absolute lymphopenia and anemia as shown by low hemoglobin, hematocrit, and RBC mass compared with WT (Figure 7). In contrast, AAV-SPL–treated mice were not anemic, whereas lymphopenia persisted despite AAV-SPL treatment.

**Figure 8:**
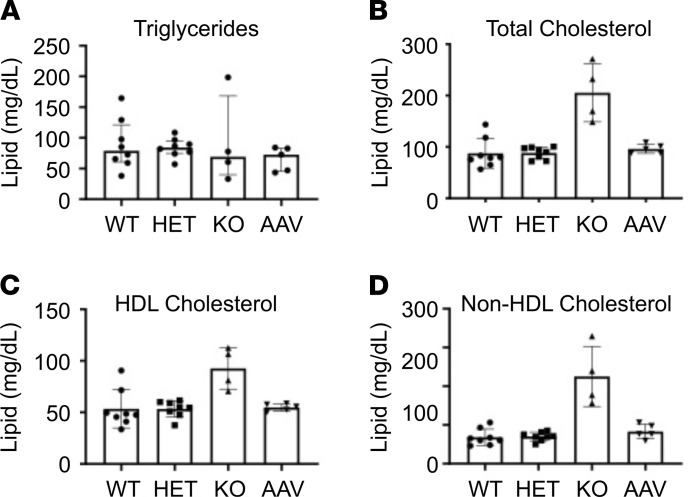
Plasma lipids of treated and untreated *Sgpl1*-KO mice. Plasma (**A**) triglycerides, (**B**) total cholesterol, (**C**) HDL-cholesterol, and (**D**) non–HDL-cholesterol (LDL, IDL, and VLDL) were measured in plasma of WT (*n* = 8), heterozygous (HET) (*n* = 8), KO (*n* = 4), and AAV-SPL–treated KO (AAV) mice, (*n* = 5). All mice were euthanized at 28 DOL. For triglycerides, 1-way ANOVA was applied, and there were NSDs between any of the groups. For all other lipids, 1-way ANOVA was used to compare WT, HET, and AAV-SPL, and unpaired *t* test was used to compare KO and WT. Comparing KO vs. WT: (**B**, *P* = 0.0006); (**C**, *P* = 0.007); (**D**, *P* = 0.0002). There are NSDs between WT, HET, and AAV-SPL for any of the lipids.

*Sgpl1*-KO mice were previously shown to exhibit high circulating cholesterol levels, including total, free, and esterified cholesterol (15). LDL- and HDL-cholesterol were also substantially increased in KO compared with WT blood. Comparing plasma of WT, HET, KO, and AAV-SPL–treated KO mice at the time of weaning, we confirmed significantly higher levels of total cholesterol, as well as higher HDL- and non–HDL-cholesterol in KO plasma compared with WT or HET plasma, whereas triglyceride levels were not appreciably different in any of the groups (Figure 8). AAV-SPL–treated mouse plasma had total cholesterol, HDL-cholesterol, and non–HDL-cholesterol levels that were no different from WT and HET controls. Therefore, the impact of SPL disruption on circulating cholesterol was completely prevented by *SGPL1* gene replacement.

Results of tests of glucose, electrolytes, and markers of liver and kidney function in the plasma of WT, KO, and AAV-SPL–treated KO mice are shown in Table 1. Normal ranges provided for BALB/c mice by the reference laboratory served as rough guidelines, considering our study utilized mice in a C57BL/6 background. *Sgpl1*-KO mice exhibited normal creatinine but low albumin and high blood urea nitrogen levels, which were corrected or partially corrected with treatment. Total bilirubin and alanine transaminase levels were within normal ranges and not significantly different among the groups. Aspartate transaminase levels were within normal limits, although untreated KO mice had significantly higher levels than WT. Interestingly, KO mice exhibited low glucose levels, consistent with the glucocorticoid deficiency observed in these mice. Glucose levels normalized with treatment in the KO group. The only abnormality that correlated specifically with AAV-SPL treatment was a high chloride level. The significance of this is not known but could theoretically be related to metabolic acidosis that might result from deficient steroid production in treated KO mice.

To determine whether AAV-SPL altered S1P gradients and thereby disrupted lymphocyte trafficking in healthy mice, treatment was administered to 3 WT newborn mice. Plasma S1P levels and whole blood counts were measured at 3 months of age. The difference in plasma S1P levels between untreated (563 ± 244 nM) and treated (588 ± 168 nM) WT mice was not significant. As shown in Supplemental Table 2 (supplemental material available online with this article; https://doi.org/10.1172/jci.insight.145936DS1), treated and untreated WT mice also did not exhibit significant differences in any hematological parameter.

### AAV-SPL is expressed and active in key target tissues.

The pattern of AAV-SPL bioavailability was investigated in *Sgpl1* WT, KO, and AAV-SPL–treated KO mice. In some cases, AAV-SPL^K353L^–treated KO mice were also included for comparison. Mice were euthanized for tissue harvest at 3 weeks of life unless otherwise stated.

**Figure 9:**
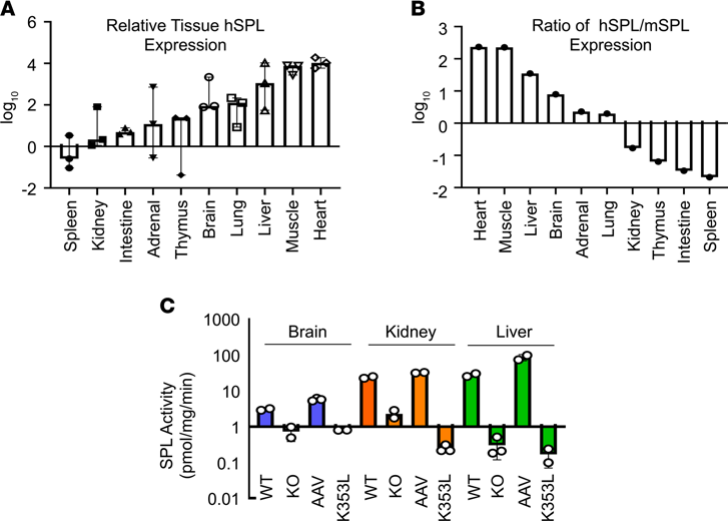
Bioavailability of AAV-SPL based on SPL gene expression and enzyme activity. (**A**) Relative hSPL (*SGPL1*) levels in different tissues of AAV-SPL–treated KO mice, shown as log_10_ values. (**B**) Ratio of hSPL (*SGPL1*) to mSPL (*Sgpl1*) in tissues of WT and AAV-SPL–treated KO mice, respectively. (**C**) SPL activity levels in brain (blue bars), kidney (red bars), and liver (green bars) of WT, KO, AAV-SPL–treated KO (AAV) and AAV-SPL^K353L^ treated KO (K353L) mice. Student’s *t* test: in all 3 tissues, for WT vs. KO, *P* < 0.03; AAV vs. KO, *P* < 0.02; KO vs. K353L, NSD. For **A**–**C**, *n* = 3 per group.

Using quantitative RT-PCR, we compared *SGPL1* expression levels in a broad range of tissues. Consistent with the known tropism of AAV9, hSPL (*SGPL1*) expression was high in heart and skeletal muscle, liver, and lung and lowest in spleen, kidney, and intestines (Figure 9A). In contrast, endogenous mouse SPL (mSPL, *Sgpl1*) expression was highest in thymus and intestine; moderate in kidney, brain, adrenal gland, and liver; and lowest in lung, spleen, and muscle, similar to our previously reported findings (data not shown and ref. 37). Representing the expression of the transgene relative to endogenous expression as a ratio, the highest levels were observed in heart, muscle, liver, and brain and lowest in thymus, intestine, and spleen (Figure 9B). Relative transgene expression in kidney, adrenal gland, brain, and liver were calculated to be 17%, 2-fold, 8-fold, and 35-fold of endogenous mSPL expression levels, respectively. As shown in Figure 9C, SPL activity levels in AAV-SPL–treated KO brain, kidney, and liver were comparable to or above WT levels, ranging from 3 to 6 pmol/mg/min in brain, 24 to 32 pmol/mg/min in kidney, and 28 to 87 pmol/mg/min in liver. SPL activity in untreated KO and AAV-SPL^K353L^–treated KO tissues ranged from 0.17 to 2.3 pmol/mg/min. Adrenal gland SPL activity could not be measured because of the limited amount of available tissue.

**Figure 10:**
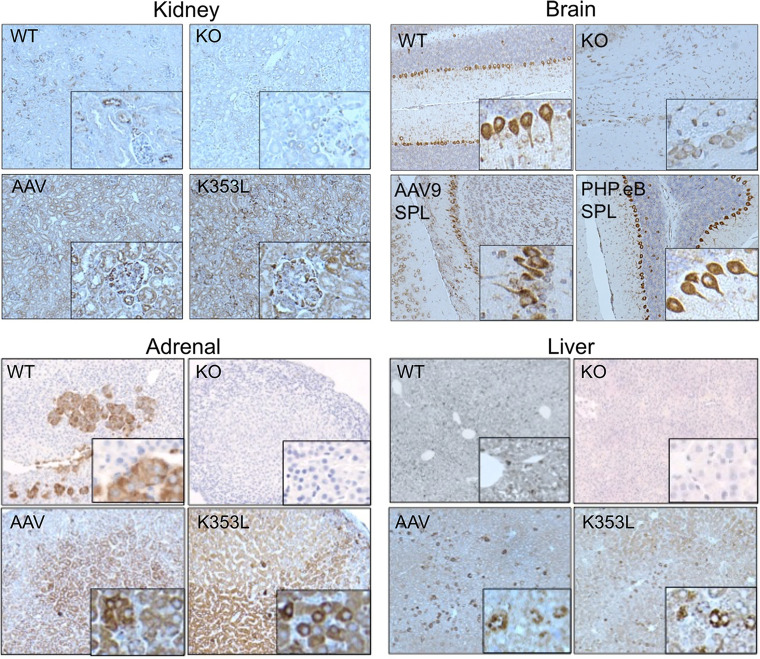
Expression pattern of mSPL and hSPL in murine tissues. IHC was performed on fixed tissue sections from WT, KO, AAV-SPL–treated KO (AAV), and AAV-SPL^K353L^–treated KO (K353L) mice. For WT tissues, staining was performed using anti-mSPL. For all other groups, staining was performed with anti-hSPL. KO tissues were also stained with anti-mSPL and were negative for signal (data not shown). Brain tissues from KO mice treated with PHP.eB-hSPL are shown in comparison with AAV9-hSPL. Insets show enlarged image detail for each quadrant to highlight cells with positive signal; original magnification, ×20.

To further confirm our findings and characterize regional transgene expression patterns within key organs affected by SPLIS, IHC was performed on fixed brain, adrenal gland, kidney, and liver sections from WT and KO and AAV-SPL– or AAV-SPL^K353L^–treated mice. As shown in Figure 10, endogenous mSPL expression was detectable in all 4 WT tissues, whereas no signal was detected in *Sgpl1*-KO tissues. In liver, mSPL expression was relatively homogeneous. In other tissues, specific cell types expressed high mSPL levels, including medullary cells of the adrenal gland, Purkinje cells of the cerebellum, and both tubular and glomerular cells in the kidney. In AAV-SPL–treated *Sgpl1*-KO mouse tissues, strong hSPL signal was detected in all 4 tissues, although in most cases the patterns were dissimilar to endogenous mSPL expression patterns, consistent with the fact that the transgene is under regulation of the CMV promoter and not the endogenous *Sgpl1* promoter. The expression pattern of hSPL in the brain was unique among the 4 tissues in that it mirrored that of the endogenous mSPL expression pattern, which was strikingly positive in Purkinje cells. This is likely a fortuitous coincidence related to the transduction pattern associated with i.v. delivery of AAV9 to pups (38). To improve delivery of AAV-SPL to brain, we packaged the virus in PHP.eB, a designer capsid with reportedly enhanced neuronal targeting capacity (39). The brain tissue of *Sgpl1*-KO mice treated with AAV-SPL packaged in PHP.eB showed a similar pattern of Purkinje cell targeting that appeared more intense than that observed in mice treated with our standard AAV9. The hSPL expression pattern in *Sgpl1*-KO pups treated with catalytically inactive AAV-SPL^K353L^ mirrored that of AAV-SPL–treated mouse tissues, as expected.

### AAV-SPL reduces plasma and tissue sphingolipids in Sgpl1-KO mice.

**Figure 11:**
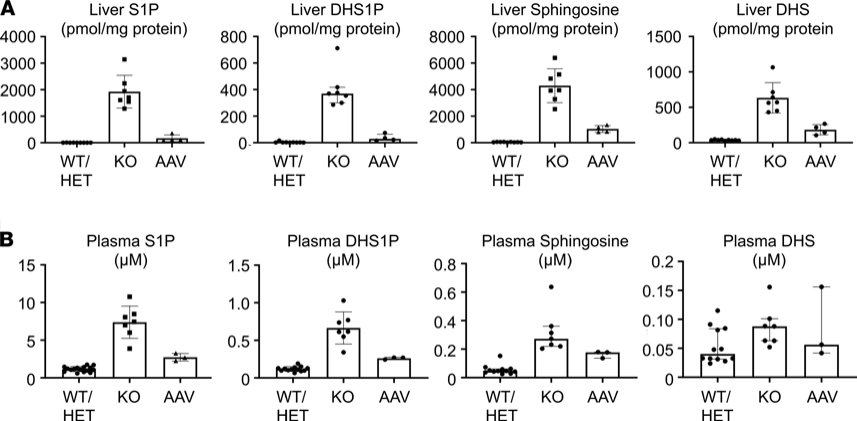
Attenuation of sphingolipid accumulation in AAV-SPL–treated *Sgpl1*-KO mice. S1P, dihydrosphingosine-1-phosphate (DHS1P), sphingosine, and dihydrosphingosine (DHS) in the (**A**) liver and (**B**) plasma of WT (*n* = 9), KO (*n* = 7), and AAV-SPL–treated KO (AAV, *n* = 4) mice. For liver S1P, liver DHS1P, and plasma sphingosine, Mann-Whitney *U* with Bonferroni’s correction was applied. For liver sphingosine, liver DHS, plasma S1P, and plasma DHS1P, unpaired *t* test with Bonferroni’s correction was applied, and for liver DHS and plasma DHS1P, Welch’s correction was additionally applied. For plasma DHS, Kruskal-Wallis was applied. For liver S1P: KO vs. WT/HET, *P* < 0.004; AAV vs. WT/HET, *P* = 0.0058; AAV vs. KO, *P* = 0.012. For liver sphingosine: KO vs. WT/HET, *P* < 0.0002; AAV vs. WT/HET, *P* < 0.0002; AAV vs. KO, *P* = 0.0016. For liver DHS1P: KO vs. WT/HET, *P* = 0.0004; AAV vs. WT/HET, *P* = 0.0112; AAV vs. KO, *P* = 0.0122. For liver DHS: KO vs. WT/HET, *P* = 0.0006; AAV vs. WT/HET, *P* = 0.0062; AAV vs. KO, *P* = 0.049. For plasma S1P: KO vs. WT/HET, *P* = 0.0002; AAV vs. WT/HET, *P* = 0.0002; AAV vs. KO, *P* = 0.014. For plasma sphingosine: KO vs. WT/HET, *P* < 0.0002; AAV vs. WT/HET, *P* < 0.0018; AAV vs. KO, *P* = 0.033. For plasma DHS1P: KO vs. WT/HET, *P* = 0.001; AAV vs. WT/HET, *P* < 0.0002; AAV vs. KO, *P* = 0.0048. For plasma DHS: there were NSDs in any of the groups.

*Sgpl1*-KO mice accumulate tissue S1P, reaching levels up to many hundred-fold higher than their WT and heterozygous littermates, most notably in liver, intestines, and heart (Table 2). Because SPL guards the exit point of the sphingolipid degradative pathway, other sphingolipids, including unphosphorylated and phosphorylated sphingoid bases, ceramides, and dihydrosphingolipids, also accumulate in *Sgpl1*-KO mouse tissues. When liver S1P levels were compared in untreated and AAV-SPL–treated *Sgpl1*-KO mice, a dramatic reduction in S1P was observed in response to treatment, although levels remained higher than those of WT (Figure 11A). Similarly, the elevated levels of dihydroS1P, sphingosine, and dihydrosphingosine (Figure 11A) as well as C14, C16, and C18 ceramides (Supplemental Figure 1) observed in KO liver were reduced by treatment. In contrast, C20 ceramides were not elevated in untreated KO liver and remained unchanged in response to treatment. Plasma S1P levels in untreated KO mice were 7-fold higher than WT levels (Figure 11B). KO plasma S1P levels were reduced by 50% in response to treatment. Similarly, the elevated levels of plasma dihydroS1P, sphingosine, and dihydrosphingosine in untreated KO mice were reduced in response to treatment.

### The effects of AAV-SPL are durable over time.

**Figure 12:**
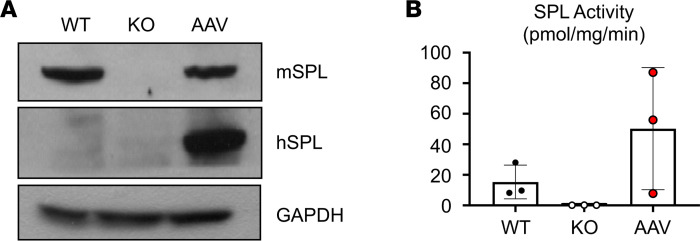
Durability of AAV-SPL. (**A**) Abundance of liver mSPL and hSPL are shown by immunoblot of livers from WT, KO, and long-lived AAV-SPL–treated KO mice (AAV). Blots were probed using anti-mSPL, which detects mSPL and crossreacts with hSPL, and using anti-hSPL, which is specific for hSPL. WT liver expresses mSPL but not hSPL. Untreated KO does not express either protein. Liver of the AAV-SPL–treated KO mouse euthanized at 11.5 months of age shows abundant hSPL, detected by both antibodies. GAPDH is a loading control. (**B**) SPL activity measured in livers of WT, KO, and long-lived AAV-SPL–treated KO (AAV) mice (*n* = 3/group) euthanized at 11 months of age. Using unpaired *t* test with Bonferroni’s correction, there were NSDs between the groups.

Importantly, hSPL protein expression persisted over time as shown by the strong SPL signal in immunoblots of liver extracts harvested from AAV-SPL–treated KO mice as late as 11 months after injection (Figure 12A). SPL enzyme activity in the liver tissues of long-lived AAV-SPL–treated KO mice was at or exceeded the level in WT mouse liver, although because of the wide variability in activity, the differences between groups did not reach statistical significance (Figure 12B). These overall results demonstrate that AAV-mediated *SGPL1* gene transfer results in SPL expression and activity in a broad range of murine tissues and is durable over time.

### Treatment of Sgpl1-KO mice with AAV-SPL elicits an immune response.

To determine whether *Sgpl1*-KO mice respond immunologically to AAV-SPL, ELISAs were performed to detect the presence of anti-AAV9 in the plasma (Supplemental Figure 2). In untreated *Sgpl1*-KO mouse plasma, anti-AAV9 antibodies were detected in the range of 150–750 ng/mL, whereas treated KO mouse plasma had a significantly higher concentration of AAV9-specific antibodies, in the range of 2000–3500 ng/mL. Anti-hSPL antibodies trended higher in the treated group, but the difference was not significant. Overall, our results demonstrate that untreated mice have low but detectable amounts of anti-AAV9 antibody and that *Sgpl1*-KO mice have a functional immune response to the AAV vector in spite of their lymphopenia.

## Discussion

SPLIS is a rare lethal childhood syndrome that was first recognized 4 years ago. Of the main disease features, SRNS leading to rapid development of end-stage renal disease and progressive neurological deterioration are responsible for most SPLIS-associated deaths, and adrenal insufficiency is also highly consequential. The characteristic lymphopenia is caused by high tissue S1P levels, which disrupt S1P chemotactic gradients critical for regulating lymphocyte egress (16, 17, 40). With the exception of the well-established role of S1P in lymphocyte trafficking, the pathomechanisms responsible for the varied phenotypes associated with SPLIS remain poorly understood. Pathology could stem from accumulation of cellular S1P or other sphingolipids, aberrant S1PR signaling, loss of SPL products, or a combination of these.

Various targeted therapeutic approaches could be considered for treating patients with SPLIS. Some SPLIS mutations produce poorly expressed mutant SPL proteins that aggregate in cultured cells, which suggests protein misfolding and rapid clearance (3). Supplementation with high-dose vitamin B_6_ (which can overcome reduced affinity for the cofactor and/or exert chaperone functions for B_6_-dependent enzymes) (41) or chemical chaperones (42) could potentially stabilize some mutant SPL proteins. There is evidence in support of this approach (10). However, these strategies would not be as effective in patients harboring *SGPL1* alleles with deletions, nonsense or catalytic site mutations, or splicing defects. Substrate depletion strategies are effective in some patients with sphingolipidoses and attenuate phenotypes observed in SPL/*Sply* mutant fruit flies (43–45). However, l-cycloserine — the only FDA-approved drug that blocks sphingolipid biosynthesis by inhibiting serine palmitoyltransferase — also inhibits SPL activity (Dominic Campopiano, University of Edinburgh, personal communication). Supplementation with SPL products could have a role in SPLIS treatment. However, SPL products hexadecenal and ethanolamine-1P are not FDA approved, and their deficiency is unlikely to cause all SPLIS disease manifestations. Sphingosine kinase inhibitors, S1P-binding monoclonal antibodies, and S1PR antagonists may have a place in SPLIS treatment strategies but will not address the consequences of ceramide accumulation and SPL product deficiency.

Gene or enzyme replacement and gene editing are the only strategies with the potential to fully restore SPL activity in all patients with SPLIS. Developing an effective enzyme replacement therapy that can deliver SPL protein to the endoplasmic reticulum or provide a surrogate membrane system would be challenging. While gene editing technology is developing rapidly, problems with off-target effects and efficient delivery in an intact organism remain to be solved. Based on these considerations and the lack of an available mouse model for testing gene editing approaches, we chose to investigate gene therapy as a promising near-term therapeutic strategy for SPLIS, a condition in which the risk/benefit ratio is likely to be acceptable.

We delivered human *SGPL1* via AAV-mediated gene transfer to newborn *Sgpl1*-KO pups, using the strong, constitutive CMV promoter/enhancer to drive expression of the transgene. The dose was chosen based on the concentration of our viral preparation and the maximum tolerated volume of an i.v. bolus that newborn pups can safely tolerate (i.e., about 20 μL). We chose to investigate the impact of our treatment on survival as a first goal, with the expectation that a dose-response experiment might be necessary to optimize any discernable impact on survival. The effects we observed in the first 10 pups were so dramatic — with one-third of the cohort living 8 to 11 months — that we were compelled to undertake further analysis of the effects of our treatment on key disease features and lipids to uncover the reasons for this auspicious outcome.

After optimization of our injection protocol, most treated mice survived for months without developing nephrosis, as shown by normal urine ACR and plasma albumin levels and no evidence of glomerulosclerosis. This would suggest that 7 × 10^14^ vg/kg represents a threshold dose needed to achieve efficacy in our model.

The STAT3 pathway was activated in the kidney parenchyma of *Sgpl1*-KO mice, as shown by Stat3 phosphorylation, upregulation of Stat3 target genes, and upregulation of *Il-6*. Importantly, this proinflammatory phenotype was prevented by treatment with AAV-SPL but not by a catalytically inactive control. STAT3 transcriptional activation has been implicated in the pathogenesis of diabetic kidney disease, Alport syndrome, lupus nephritis, nephrotoxic nephritis, and polycystic kidney disease (34). In addition, STAT3 activation has been shown to promote the progression of focal segmental glomerulosclerosis, which is manifest in patients with SPLIS (46–48). One of the many activators of the Stat3 pathway is S1PR-mediated signaling (30, 31, 49). We found previously that gut-specific disruption of murine *Sgpl1* results in sensitivity to colitis due to Stat3 activation (50). Thus, we suspected activation of Stat3 and resulting inflammation might underlie the nephrotoxic consequences of global *Sgpl1* disruption. Our findings suggest that STAT3 represents a critical nexus between S1P signaling and kidney injury. While beyond the scope of this study, further investigation of the role of STAT3 in SPLIS and the intersection of sphingolipids, STAT3, and kidney disease is clearly warranted.

A battery of neurobehavioral tests designed to measure the normal acquisition of key developmental milestones of murine pups from birth to weaning was used to compare the developmental progress of untreated and treated *Sgpl1-*KO pups and WT littermate controls. Our results revealed the delayed achievement of 6 milestones in untreated KO pups, consistent with the neurological and behavioral defects observed in brain-specific *Sgpl1*-KO mice (26). In contrast, treated KO pups showed no evidence of developmental delay. A detailed study to monitor motor and behavioral function of treated *Sgpl1*-KO mice that reach adulthood will be required to establish the full impact of AAV-SPL on neuronal function. Optimization of brain targeting, such as by intrathecal or intracerebral delivery or using other capsid types that efficiently target neurons, may be required to maximize the impact of treatment on the CNS.

Our results show for the first time to our knowledge that *Sgpl1*-KO pups exhibit corticosterone deficiency. Consistent with a state of glucocorticoid insufficiency, plasma glucose levels of KO pups were at the low end of the normal range and significantly lower (*P* = 0.04) than those of WT pups. While plasma ACTH levels trended lower in KO compared to WT mice, these trends were not statistically significant. Further study will be required to fully characterize the adrenal cortical defect, the hypothalamic-pituitary-adrenal axis, and the function of other endocrine organs to establish the origin and extent of the endocrine defect. Treatment with AAV-SPL did not prevent corticosterone deficiency in our study. Interestingly, the relative hypoglycemia of the KO pups was corrected with AAV-SPL treatment. This finding suggests the possibility that glucocorticoid deficiency was in fact corrected by treatment but that we failed to detect this because of a sampling error or an insufficient number of subjects in our analysis.

Additional phenotypes reported in *Sgpl1*-KO pups, including anemia, high circulating cholesterol levels, and high Th-17 and other cytokines, were confirmed in our study. In addition, we observed elevated levels of the profibrogenic cytokine *Tgf-β* in *Sgpl1*-KO mouse tissues. These phenotypes were reduced or completely prevented by AAV-SPL treatment. The complete responsiveness of hypercholesterolemia to AAV-SPL may be due to the high transduction efficiency of the liver with AAV9 combined with the liver representing the main site of cholesterol biosynthesis. On the other hand, lymphopenia persisted in treated KO mice, consistent with the known sensitivity of lymphocyte trafficking to modest increases in circulating S1P. While this is a limitation of our treatment, immunological rescue may not be necessary. Despite lymphopenia in patients with SPLIS, T cell function persists, and response to childhood vaccines is usually present (3, 10). AAV-SPL–treated KO mice mounted an immune response to both AAV and the transgene, indicating their immune system is functional despite their lymphopenia. Similarly, mice lacking SPL specifically in bone marrow are healthy, with normal life spans despite their lymphopenia (17). If necessary, systemic gene therapy could be combined with bone marrow transplantation, as has been reported for other genetic disease models (51).

The high tissue levels of S1P and upstream sphingolipid intermediates in the livers of untreated *Sgpl1*-KO pups were substantially reduced in response to treatment, although the levels remained well above those of WT controls. Similarly, plasma S1P and other circulating sphingolipids were at least 50% lower in treated versus untreated KO mice. These findings demonstrate that tissue SPL activity in response to AAV-SPL treatment markedly attenuated the biochemical derangements characteristic of SPLIS.

Human SPL was widely expressed in treated KO mice, being detectable in both tubular and glomerular cells of the kidney, medullary cells of the adrenal gland, and Purkinje cells of the cerebellum. The transgene was expressed at 17% of endogenous levels in the kidney, but this may suffice, considering that podocytes, the key cells responsible for the blood filtration function of the kidney, make up only 3% of total kidney mass. The highly specific pattern of hSPL expression in cerebellar Purkinje cells mirrored that of endogenous mSPL, which is unexpected from a CMV promoter–driven transgene but can be explained by the coincidental Purkinje cell–specific cerebellar expression pattern of AAV9 after i.v. delivery to newborn mice (38). Importantly, expression in liver persisted for up to 11 months in long-lived mice, demonstrating the durability of the effect. The expression pattern was consistent with the effect on specific phenotypes, including protection against nephrosis, neurological defects, and inflammatory signaling in the liver. Use of genetically engineered capsids may further enhance site-specific and temporal transgene expression. For example, rationally designed artificial capsids such as Anc80 and PHP.eB may improve delivery to kidney and neuronal tissues, respectively (39, 52).

There are several limitations to our study. A single AAV-SPL dose was used in the entire experiment, so we cannot identify a minimal effective dose. We chose AAV9 to package the virus because of its broad tropism. Despite our promising bioavailability results, the potential for different capsids to achieve still higher expression remains. We chose the first days of life as a single treatment time point. It remains possible that administration of virus at a later age, but still before irreversible organ damage occurs, would increase transduction efficiency and efficacy.

Our analysis of neurodevelopment was hampered by the inability of the operator to maintain blindness to genotype because untreated *Sgpl1*-KO pups are runted, which becomes obvious after the first week of life. Reliance on highly objective milestones along with the presence of 2 observers at each testing period were employed to minimize observer bias.

Anti-AAV antibodies were detected in treated mice. Their presence could present challenges to optimization and translation to the clinic, considering that 30% of patients have anti–capsid 9 antibodies, causing them to be excluded from clinical trials with AAV9. Further, gene therapy could elicit an immune response to the transgene, especially in patients with SPLIS who produce no SPL protein. These challenges could be overcome by using endopeptidase to eliminate capsid-specific antibody, as recently described (53).

The high ectopic expression of SPL we achieved by expressing *SGPL1* under the control of the CMV promoter could have undesirable effects, such as the susceptibility of the CMV regulatory sequence to silencing, potential integration effects including carcinogenesis, and toxicity of ectopic SPL expression. Substituting an alternate promoter could lead to improved efficacy, bioavailability, and safety compared with the CMV promoter.

We included both sexes in our study, and both male and female mice were among the longest lived animals. However, a larger study will be required to discern any potential sex-specific differences in efficacy and effects on male and female reproduction.

Last, the *Sgpl1*-KO mouse — while a robust model of severe SPLIS — may not faithfully emulate the pathologies found in SPLIS forms caused by mutations that do not eliminate SPL entirely. In the future, the development of murine models harboring missense mutations created through gene editing technologies would afford additional systems for testing therapeutic strategies employing gene replacement and repair.

In aggregate, our findings demonstrate that AAV-SPL treatment delivered early in life can avert the development of nephrosis, neurobehavioral deficits, lipidosis, and aberrant inflammatory signaling, enabling long-term survival in a mouse model of SPLIS. The efficacy of AAV-SPL is consistent with the broad expression pattern and enzyme activity observed in mice treated with AAV-SPL. Our preclinical results suggest that *SGPL1* gene replacement holds promise as a universal targeted treatment for SPLIS. Safety studies and human clinical trials will ultimately reveal the translational relevance of our auspicious initial results.

## Methods

### Reagents.

We used S1P, hexadecenal(d5), and Ceramide/Sphingoid Internal Standard Mixture I (Avanti Polar Lipids); 2-hydrazinoquinoline (MilliporeSigma); and HPLC vials (Filtrous).

### Vector production and packaging.

Human WT *SGPL1* cDNA, hSPL^K353L^ cDNA, and hSPL-tRFP were separately subcloned into EcoRI/XhoI sites in pAAV-MCS (Agilent Technologies). Inserts were confirmed by DNA sequencing. AAV was packaged with different capsids to generate AAV8-, AAV9-, and AAV-PHP.eB-SPL virus. AAV was packaged using an adenovirus-free system in which AAV-SPL, pHelper, and AAV-RC plasmid pUCmini-iCAP-PHP.eB (Addgene plasmid 103005) were cotransfected into HEK293 cells (ATCC). Virus was harvested and purified via iodixanol gradient ultracentrifugation (54). After validating activity, AAV9-hSPL, PHP.eB-hSPL, and AAV9-hSPL^K353L^ virions were prepared at large scale for in vivo use. Virus titers were quantified by Taqman quantitative PCR, and probes were determined to be ~3.5 × 10^10^ vg/μL.

### Functional testing.

Virus particles expressing WT hSPL, hSPL, and RFP, or catalytically inactive mutant hSPL^K353L^, were used to infect immortalized fibroblasts derived from a patient with SPLIS (10). Fibroblasts were generated and cultured in DMEM with 10% FBS as described previously (55). AAV9-hSPL and AAV9-hSPL-tRFP viruses were used to transduce fibroblasts. After 48 hours, cells were harvested for analysis.

### Immunoblotting.

Proteins were extracted as described (10). Antibodies used were goat anti-human *SGPL1* (AF5535, R&D Systems, Bio-Techne), anti-mouse *Sgpl1* (56), rabbit anti-GAPDH (sc-25778, Santa Cruz Biotechnology), anti–phosphorylated Stat3 (9145, Cell Signaling Technology), and anti–total Stat3 (9131, Cell Signaling Technology). HRP-conjugated secondary antibodies [115-035-003, Goat Anti-Mouse IgG (H+L); 111-035-144, Goat Anti-Rabbit IgG (H+L), Jackson ImmunoResearch; sc-2020, donkey anti-goat IgG-HRP, Santa Cruz Biotechnology] were used to detect signal using SuperSignal West Pico kit (Thermo Fisher Scientific). Radiographic bands were quantified using NIH ImageJ.

### SPL assays.

SPL activity was quantified by measuring formation of (2*E*)-hexadecenal(d5) by quantification of its hydrazine derivative as described (57).

### Animals.

SPL-KO mice, bred for over a decade in the UCSF animal facility, exhibit no SPL expression or activity and accumulate sphingolipids (15). The KO allele was generated using the ROSAFARY gene trap vector (24, 58). Heterozygous mice are viable and fertile and are crossed to obtain homozygous progeny. The KO is born at 25% frequency. The line was backcrossed to C57BL/6 background for 10 generations. *Sgpl1* heterozygous KO mice were maintained in an Association for Assessment and Accreditation of Laboratory Animal Care International–accredited animal facility with an automated light/dark cycle of 7 am/7 pm. Pups were genotyped by toe biopsy using primers: SPL-F: CGCTCAGAAGGCTCTGAGTCATGG, SPL wt-R: CCAAGTGTACCTGCTAAGTTCCAG, and SPL ko-R: CATCAAGGAAACCCTGGACTACTG.

### AAV treatments.

Homozygous KO pups were anesthetized with isoflurane and injected with virus (3.5–7.0 × 10^11^ vg) in 10–20 μL sterile saline into the superficial temporal vein using a 33-gauge needle and Yale model YA-12 syringe pump. To aid temporal vein injection, the vector solution contained 1% food coloring. In some cases, virus solution was instead injected into the liver parenchyma as described (59). Injected mice were monitored and weighed every 3 days and subjected to 24-hour urine collections in a metabolic chamber, serial neurobehavioral examinations, and/or phlebotomy at various time points. Survival study mice were euthanized when they reached the humane endpoint. Other mice were euthanized at 21–28 DOL.

### Laboratory analyses.

Serum and urine albumin and creatinine were measured using the COBAS INTEGRA 400 plus instrument (Roche Diagnostics) by the University of California Davis Comparative Pathology Laboratory (Davis, California, USA).

### Kidney pathology.

Periodic acid–Schiff staining of 3 μM sections of FFPE mouse kidneys was performed. Glomerular tuft area (which includes capillaries, mesangium, and podocytes) was measured across 30 glomeruli from each mouse kidney, sampling the full depth of the cortex, using QuPath quantitative pathology and bioimage analysis software after scanning sections stained with Periodic acid–Schiff to whole-slide images with a Leica AT2 scanner (Aperio).

### RNA isolation and mRNA analysis.

Total RNA was extracted by TRIzol reagent (Thermo Fisher Scientific). DNase was used to remove genomic DNA contamination as described (60). A total of 4 μg of DNase I–treated RNA was used for the first-strand cDNA synthesis by using the SuperScript III reverse transcriptase to synthesize reverse transcription products (Life Technologies, Thermo Fisher Scientific). Quantitative real-time PCR was performed by using PowerUp SYBR Green Master Mix in QuantStudio 12K Flex Real-Time PCR System (Life Technologies, Thermo Fisher Scientific). Primers used are listed in Supplemental Table 1.

### Neurological examination of preweaned mice.

A battery of neurodevelopmental tests for preweaned mice was developed in accordance with existing protocols (61–64). Mice were assessed for achievement of bilateral eye opening starting on DOL 11, hearing onset starting on DOL 11, adult walking pattern starting on DOL 3 grip strength starting on DOL 21, and cliff aversion reflex starting on DOL 3. The walking pattern was also evaluated on DOL 9, and pups were given a score of 0 for nonlinear pivoting, 1 for mixed ambulation, 2 for fully linear movement. Each mouse underwent 3 sets of grip strength tests using a Chatillon Ametek digital force gauge model DFIS-10. Cliff aversion reflex was marked as present or absent daily across 8 days of testing. A score was assigned to each pup by summing the number of days that the reflex was present, resulting in a score from 0 to 8. Results were aggregated across the 4 groups. Two operators were present at each testing session.

### Corticosterone and ACTH measurements.

Corticosterone was measured by radioimmune assay (RIA), using a commercial kit (MP Biomedicals) at the University of Virginia Ligand Assay & Analysis Core (Charlottesville, Virgina, USA). The method was validated for mouse serum using a protocol based on recommendations of the Endocrine Society’s Sex Steroid Assays Reporting Task Force (65). The RIA evaluation included the following indices: accuracy, linearity, functional sensitivity, precision, and correlation to a previous or established method. Assay characteristics were as follows: sensitivity = 15 ng/mL; intra-assay coefficient of variation (CV) = 6.3%; inter-assay CV = 7.1%. ACTH was measured using the Abcam Mouse/Rat ACTH ELISA Kit, according to the manufacturer’s instructions.

### Cholesterol measurements.

Plasma triglycerides, total cholesterol, and HDL-cholesterol were measured by enzymatic endpoint analysis using enzyme reagent kits (AMS Alliance) on a clinical chemistry analyzer (Liasys 330, AMS Alliance).

### IHC.

IHC was performed essentially as described (37). FFPE tissues were deparaffinized and incubated for 30 minutes in 3% hydrogen peroxide/methanol to quench endogenous peroxidases. Sections were rinsed in PBS and immunostained with anti-mSPL antisera at 1:200 dilution in 0.5% PBS/OVA at 37°C for 1 hour after antigen retrieval with citrate buffer (pH 6.0) in an autoclave set for 125°C for 2 minutes; slides were cooled for 1 hour before adding secondary antibody. Secondary antibody was biotinylated anti-rabbit (PV6121, Leica Biosystems) diluted 1:1000 in 0.5% PBS/OVA and incubated for 30 minutes at room temperature. Sections were incubated with VECTASTAIN Elite ABC kit (Vector Laboratories, Maravai LifeSciences) for 30 minutes and rinsed in PBS. Detection was performed with DAB (Vector Laboratories, Maravai LifeSciences) for 2 minutes and counterstained in hematoxylin.

### Sphingolipid profiling.

Plasma was obtained from anticoagulated whole blood by subjecting it to centrifugation at about 250*g* for 10 minutes at 4°C, followed by collection of plasma, which was stored at –80°C until analysis. To extract lipids, frozen tissues were bead homogenized in Tris buffer using a FastPrep FP120 cell disrupter (Thermo Fisher Scientific). Plasma and tissue homogenates were spiked with internal standard and extracted as described (66). Internal standard mixture contained sphingosine, C17:0 ceramide, D-*erythro*-sphingosine-d7-1-P, C12:0 ceramide-1-P, sphingomyelin (d18:1/C12:0), and lyso-sphingomyelin (d17:1) (Avanti Polar Lipids). Targeted metabolomics was performed using single reaction monitoring with an Agilent Technologies 6490 triple-quadrupole LC-MS/MS instrument coupled with a 1290 Infinity UPLC system as described (66).

### Detection of antibodies by ELISA.

For detection of anti-AAV9, 17 μL of AAV at 1.17 × 10^13^ vg/mL was added to 10 mL of coating buffer (100 mM carbonate/bicarbonate buffer containing 3.03 g Na_2_CO_3_, 6.0 g NaHCO_3_ per liter, pH 9.6). A total of 100 μL of this solution was used to coat each well of a 96-well plate (high-binding polystyrene Immulon 2 HB plate). The plates were left at 4°C overnight and the next day were washed 4 times with 200 μL TBS-Tween 20 (TBST) per well. Next, 200 μL of 2% BSA in TBST was used to block the wells overnight at 4°C, followed by washing. Anti-AAV9 mouse monoclonal antibody (MilliporeSigma clone HL2372, catalog MABF2309) was used to establish a standard curve. Plasma from 3 untreated and 3 AAV-SPL–treated mice was diluted 1:100 and 1:500, and 100 μL of each dilution was added to each well in triplicate. All dilutions were done in 2% BSA in TBST. Incubation was at 4°C overnight followed by washing. The following day, 100 μL of 1:4000 dilution of an alkaline phosphatase–conjugated goat anti-mouse antibody (115-055-003, Jackson ImmunoResearch Laboratories) diluted in 2% BSA in TBST was added to each well. The plate was incubated for 1 hour at room temperature, then washed 4 times with TBST. A solution of 1 mg/mL of pNPP-disodium hexahydrate (bioWORLD 21530110-4) made in a buffer containing 100 mM Tris at pH 9.5, 100 mM NaCl, and 5 mM MgCl_2_ was added at 100 μL per well to develop the ELISA at 37°C for 20–40 minutes. A plate reader was used to measure the absorbance at 405 nM, and results are reported as ng/mL. For detection of anti-hSPL, 200 ng of human SGPL1 recombinant protein (Abnova catalog H00008879-P01) was used to coat each well. The following day, 280 μL/well of TBST containing 5% fat-free milk was used to block the plate overnight. Twenty hours later, plasma samples were diluted 1:100 in TBST containing 2% BSA, and 100 μL was added to each of triplicate wells. Serial dilutions of polyclonal mouse anti-human SGPL1 antibody (Abnova catalog H00008879-A01) were added to separate wells as a positive control. The development of the ELISA plate was as for anti-AAV9 detection. Anti-hSPL is reported in arbitrary units.

### Statistics.

For comparison of 2 groups with normally distributed data, an unpaired *t* test was performed. For variances with *P* < 0.05, Welch’s correction was applied. For comparison of more than 2 groups with normally distributed data, 1-way ANOVA was performed. For skewed data, the Mann-Whitney *U* test was applied when 2 groups were compared, and Kruskal-Wallis was applied when more than 2 groups were compared. When multiple comparisons were made, Bonferroni’s correction was performed. Two-tailed *t* tests were performed in all cases. For all analyses, *P* < 0.05 was considered significant. Normally distributed data are presented as mean ± standard deviation. Skewed data are represented as median ± the interquartile range.

### Study approval.

Human skin fibroblasts were generated from skin biopsy samples obtained with informed consent in accordance with a Benioff Children’s Hospital Oakland–approved IRB protocol. Written informed consent was received from participants prior to inclusion in the study. All mouse studies were conducted in accordance with an approved University of California San Francisco IACUC protocol.

## Author contributions

JDS directed the study, conducted data analysis, and wrote the manuscript. PZ contributed to study design and performed most experiments and data analysis. GBT delivered AAV vectors to mice and performed phlebotomy and tissue harvest. GLW contributed to interpretation of results and provided critical insights. DPM and FT performed and analyzed quantitative RT-PCR studies. JYL performed SPL assays and sphingolipid measurements. JBH, JL, and YY performed and analyzed kidney pathology. BO performed immunoblotting and ELISA. JYW performed neurodevelopmental milestone assays. SK and RMK conducted cholesterol and triglyceride measurements. NK performed statistical analyses and data graphing.

## Supplementary Material

Supplemental data

## Figures and Tables

**Table 1:**
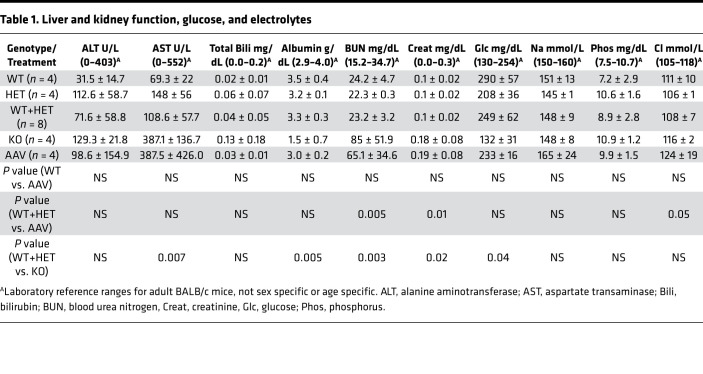
Liver and kidney function, glucose, and electrolytes

**Table 2:**
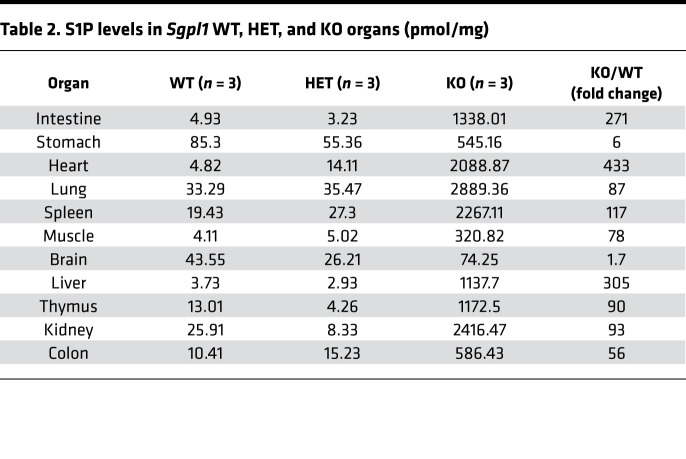
S1P levels in *Sgpl1* WT, HET, and KO organs (pmol/mg)

## Version 1 03/23/2021

In-Press Preview

## Version 2 04/22/2021

Electronic publication
