# Supplementary material for: Efficacy of AAV9-mediated SGPL1 gene transfer in a mouse model of S1P lyase insufficiency syndrome
Source: JCI Insight. 2021 Apr 22;6(8):e145936. doi: 10.1172/jci.insight.145936 (PMC8119223; doi:10.1172/jci.insight.145936)
Supplement: Supplemental data [file jciinsight-6-145936-s173.pdf]

Supplementary Materials
for Zhao et al,
"Long-term survival in a mouse model of S1P lyase insufficiency syndrome (SPLIS) after treatment with AAV9-mediated *SGPL1* gene transfer"

1. Supplementary Table 1. qRT-PCR primers used in this study
2. Supplementary Table 2. Hematological parameters in WT mice treated with AAV-SPL
3. Supplementary Figure 1. Liver ceramide levels in AAV-SPL treated *Sgpl1* KO mice
4. Supplementary Figure 2. Antibody responses to AAV9 and hSPL in treated and untreated *Sgpl1* KO mice

Supplementary Table 1. qRT-PCR primers used in this study

Primer Name	Primer Sequence
hSPL-forward	CAA GAC CAA GGA TGA TAT TAG C
hSPL-reverse	CAG AAG GCG TCC ATA GAG
mGAPDH-forward	ACC TGC CAA GTA TGA TGA
mGAPDH-reverse	GGA GTT GCT GTT GAA GTC
mSPL-forward	CTC CGA CCT GTC CTG ATT
mSPL-reverse	TAA CTG CTT CCT GCC TGA T
mMCP1 Fwd	TTAAAAACCTGGATCGGAACCAA
mMCP1 Rev	GCATTAGCTTCAGATTTACGGGT
mSPL Fwd	CTGAAGGACTTCGAGCCTTATTT
mSPL Rev	ACTCCACGCAATGAGCTGC
mLCN2 Fwd	TGGCCCTGAGTGTCATGTG
mLCN2 Rev	CTCTTGTAGCTCATAGATGGTGC
mSOCS1 Fwd	CTGCGGCTTCTATTGGGGAC
mSOCS1 Rev	AAAAGGCAGTCGAAGGTCTCG
mSOCS3 Fwd	CCCTTGCAGTTCTAAGTTCAACA
mSOCS3 Rev	ACCTTTGACAAGCGGACTCTC
mTIMP1 Fwd	GCAACTCGGACCTGGTCATAA
mTIMP1 Rev	CGGCCCGTGATGAGAAACT
mTNF alpha Fwd	CAGGCGGTGCCTATGTCTC
mTNF alpha Rev	CGATCACCCCGAAGTTCAGTAG
mIL1b Fwd	TTCAGGCAGGCAGTATCACTC
mIL1b Rev	GAAGGTCCACGGGAAAGACAC
mIFN gamma Fwd	ACAGCAAGGCGAAAAAGGATG
mIFN gamma Rev	TGGTGGACCACTCGGATGA
mTgfb-1 Fwd	CCACCTGCAAGACCATCGAC
mTgfb-1 Rev	CTGGCGAGCCTTAGTTTGGAC
mIL6 Fwd	TAGTCCTTCCTACCCCAATTTCC
mIL6 Rev	TTGGTCCTTAGCCACTCCTTC

Supplementary Table 2. Hematological parameters in WT mice treated with AAV-SPL

Genoytpe-Treatment	WBC (K/ul)	Absolute Neutrophil cells (K/ul)	Absolute Lymphocyte cells (K/ul)	Absolute Monocyte cells (K/ul)	Absolute Eosinophil cells (K/ul)	Absolute Basophil cells (K/ul)	Neutrophil %	Lymphocyte %	Monocyte %	Eosinophil %	Basophil %	RBC (M/ul)	Hemoglobin (g/dL)	Hematocrit %	MCV (fL)	MCH (pg)	MCHC (g/dL)	RDW %	Platelets (K/uL)	MPV (fL)
WT-hSPL	11.84	2.39	8.59	0.73	0.11	0.03	20.17	72.57	6.13	0.89	0.25	10.78	14.6	46.7	43.3	13.5	31.3	19.4	1033	5.9
WT-hSPL	13.18	3.50	8.75	0.82	0.11	0.01	26.52	66.38	6.21	0.85	0.04	9.43	13.1	41.8	44.3	13.9	31.3	19.8	1242	5.9
WT-hSPL	14.64	3.99	9.41	0.60	0.50	0.15	27.22	64.25	4.09	3.43	1.01	10.09	14.7	44.1	43.7	14.6	33.3	20.1	644	6.2
WT	11.30	2.58	7.92	0.65	0.13	0.03	22.80	70.07	5.76	1.11	0.25	9.73	13.2	42.2	43.4	13.6	31.3	19.4	984	5.7
WT	12.84	2.92	8.81	0.77	0.27	0.06	22.77	68.61	6.02	2.13	0.48	10.57	14.8	45.3	42.9	14.0	32.7	19.6	901	5.9
WT	11.16	2.59	7.41	0.60	0.45	0.11	23.24	66.38	5.38	4.03	0.98	9.59	13.2	39.5	41.2	13.8	33.4	17.9	656	5.7
HET	14.32	3.97	9.06	0.74	0.41	0.13	27.72	63.29	5.19	2.86	0.94	10.22	14.6	44.9	43.9	14.3	32.5	19.1	902	6.5
HET	12.80	2.65	9.22	0.71	0.19	0.03	20.67	72.06	5.57	1.50	0.20	9.95	13.8	41.8	42.0	13.9	33.0	19.5	897	6.2
HET	15.54	3.76	10.80	0.69	0.25	0.04	24.22	69.48	4.43	1.63	0.25	10.61	15.3	46.3	43.6	14.4	33.0	19.2	1035	6.3

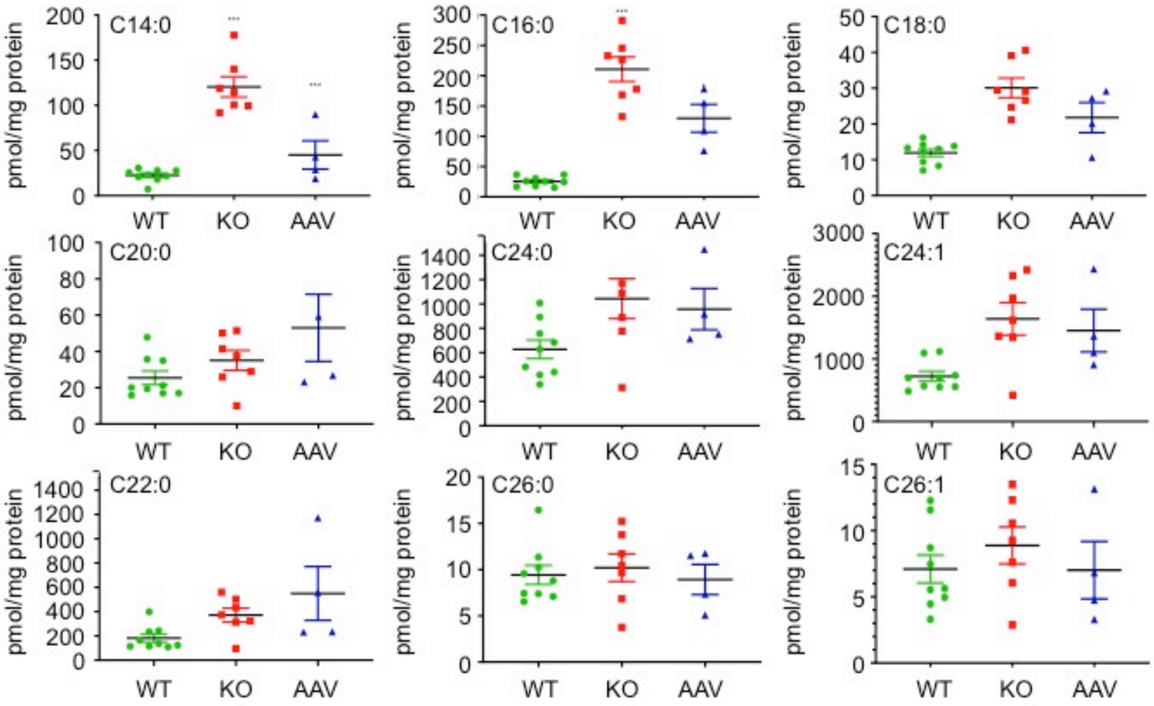

Supplementary Figure 1. Liver ceramide levels in AAV-SPL treated *Sgpl1* KO mice. Shown are the levels of different ceramide species measured by LC-MS/MS in the liver tissues of untreated wild type (WT; n=9), untreated *Sgpl1* knockout (KO; n=7) and AAV-SPL treated KO (AAV; n=4) mice. Ceramides are labeled with respect to fatty acid chain length and saturation. For C14:0: WT *vs.* KO, $p < 1 \times 10^{-5}$; WT *vs.* AAV, NSD; KO *vs.* AAV, $p < 0.008$. For C16:0: WT *vs.* KO, $p < 1 \times 10^{-5}$; WT *vs.* AAV, $p < 0.02$; KO *vs.* AAV, $p < 0.02$. For C18:0: WT *vs.* KO, $p < 0.003$; WT *vs.* AAV, no significant difference (NSD); KO *vs.* AAV, NSD. For C22:0: WT *vs.* KO, $p < 0.02$; WT *vs.* AAV and KO *vs.* AAV, NSD. For C24:0: WT *vs.* KO, $p < 0.05$; WT *vs.* AAV and KO *vs.* AAV, NSD. For C20:0, C26:0, C24:1 and C26:1, NSD for all comparisons.

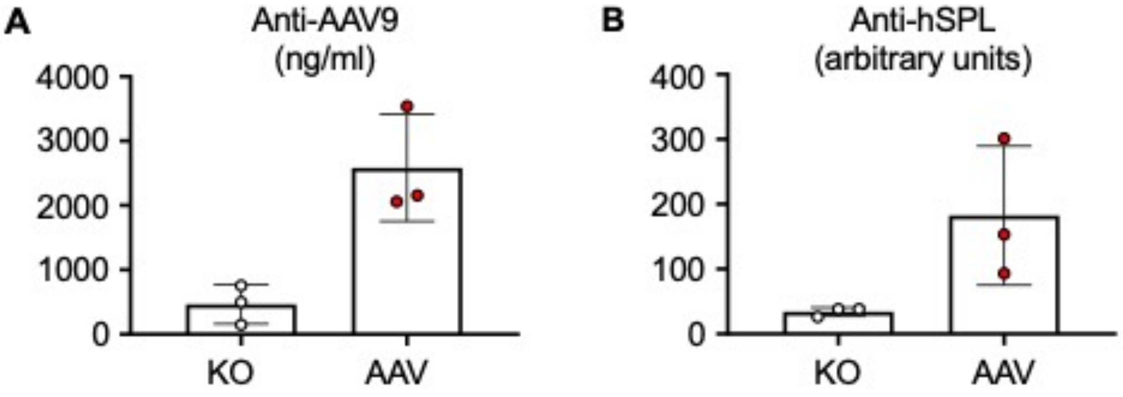

Supplementary Figure 2. Antibody responses to AAV9 and hSPL in treated and untreated *Sgpl1* KO mice. (A) Anti-AAV9 antibodies detected in the plasma of untreated (KO) and AAV-SPL treated (AAV) *Sgpl1* KO mice (n = 3/group). Anti-AAV9 mouse monoclonal antibodies of known concentration were tested by ELISA against AAV9 virus over a range of concentrations to establish a standard curve. Results are reported in ng/ml. Using unpaired t test, for KO *vs.* AAV, $p = 0.014$. (B) Anti-hSPL antibodies detected in the plasma of KO and AAV mice. Anti-hSPL mouse monoclonal antibodies in the form of ascites of unknown concentration were used in serial dilutions with purified hSPL protein to confirm antibody specificity. Antibody in plasma samples could not be quantified in absolute values and is reported in relative absorbance units. For B, KO *vs.* AAV, there was no significant difference.
